# Parents’ perceptions of school recess policies and practices

**DOI:** 10.1186/s12889-022-13831-4

**Published:** 2022-08-19

**Authors:** Isabella Ozenbaugh, Janelle Thalken, Sam Logan, Megan B. Stellino, William V. Massey

**Affiliations:** 1 Oregon State University, College of Public Health and Human Sciences, School of Biological and Population Health Sciences, Women’s Building, Room 022, 160 SW 26th Street, Corvallis, OR 97330 USA; 2 University of Northern Colorado, Social Psychology of Sport and Physical Activity, School of Sport and Exercise Science, Greeley, USA

**Keywords:** School-based recess, Parents, And disability

## Abstract

**Background:**

Previous research has shown that school recess can provide children with physical, social and cognitive benefits; yet, recess opportunities and experiences may be different for different groups of children, specifically for children living in lower income environments, children of different racial groups other than white, and for children with disabilities. Parent perceptions of recess are important to consider as they serve as advocates for their children’s access and opportunities at school as well as an additional informant for children’s experiences at recess that may be useful for policymakers and school boards to consider.

**Objective:**

To examine parent perceptions of recess by children’s disability status, children’s race and ethnicity, and family household income.

**Method:**

Participants included 473 parents from the U.S.A. stratified across six household income levels. Data were collected through an online survey using Prolific in May of 2020]. Confirmatory factor analyses were run for measures assessing parents’ perception of belonging and victimization at recess, recess policies, and recess procedures. Regression analyses were run to examine if parents’ perception of recess were predicted by race, income, or child disability status.

**Results:**

Results revealed that parents’ perceptions of recess were predicted by child disability status but not race or income. Specifically, parents’ perceptions were significantly predicted by child disability status regarding victimization (*b* = .13, *SE* = .06, *p* = .05), recess policies about withholding recess (*b* = .171, *SE* = .07, *p* = .01), and finally, student engagement at recess (*b* = .165, *SE* = .07, *p* = .02).

**Conclusion:**

Results show that parents of children with a disability perceive a different recess experience for their child that involves more instances of victimization compared to parents of typically developing children. Based on these findings, school, district, and state policy makers could consider ensuring that recess includes multiple activities, is supervised by adults, and is a space where conflict resolution occurs, for creating a more inclusive environment for children with disabilities.

## Introduction

Recess in elementary school provides children discretionary time during the day in which they can play, socialize with their peers, and be physically active. Researchers have studied the recess environment and found numerous benefits for children’s physical, cognitive, social and emotional development [1, 2] making this an important context to support public health. The growing evidence of the benefits substantiates the importance of recess time during the school day, however there are disparities in who has access to recess across the globe, and particularly within the United States of America (U.S.A) [3–6].

Since 2000, nearly 40% of school districts in the U.S.A have decreased, or eliminated, daily recess [7]. In contrast, only five states have laws that require daily recess in elementary schools [8]. The ongoing reductions in recess across the U.S.A, concurrent with the lack of policy guiding access to recess, have created an opportunity gap, which can be defined by three primary components: (1) whether children have access to daily recess; (2) whether recess is withheld for academic or behavioral reasons; and (3) if the school has taken steps to ensure that all children have access to a safe and inclusive environment for recess [4].

Researchers have further identified that children’s access to daily recess and experiences at recess may differ based on several student characteristics and demographic factors. For example, children who are from lower income families or those that attend urban schools are less likely to have access to daily recess, as well as fewer minutes of recess when compared to more affluent peers and those that reside in suburban and rural environments [3–5]. Moreover, data show that the quality of playground environments differ across various socio-economic status (SES) groups. For example, Van Dyke and colleagues reported that low SES schools were less likely to have blacktops and tracks and children were less likely to be given access to equipment during recess [9]. Researchers have also reported that principals in lower SES schools report a lack of trained supervisors at recess when compared to higher SES ﻿schools﻿ [10]. Similarly, additional data show that Black and Hispanic students are more likely to have no recess or minimal access to recess [3, 5, 6] and may be more likely to have recess withheld for academic or behavioral reasons compared to other white students [4]. Researchers have also reported that the intersection of contextual factors affecting low SES and racially and ethnically minoritized students can influence behavior on the playground. For example, Massey and colleagues reported that at low SES schools with a high percentage of racial and ethnic minority children, a lack of adequate adult supervision, and a lack of access to equipment at recess, there were, on average, more than one conflict per minute during recess [11].

Emerging research has also shown disparities at recess for children with disabilities. While children may have a primary disability within one category (e.g., development, physical, emotional), it is important to keep in mind that children may have disabilities in more than one category, or that their disability may span two categories (e.g., cerebral palsy would be both a developmental and physical disability). In considering opportunities for children with disabilities, legislation mandates the same access to recess as children without disabilities (i.e., Section 504 of the Rehabilitation Act of 1973), as well as accessibility to playground spaces (i.e., Americans with Disabilities Act), yet many students with disabilities are still being served in restrictive, segregated learning environments [12].

Children with disabilities do not always encounter recess in the same way as their peers without disabilities. Children with disabilities often experience more instances of social exclusion and bullying during recess compared to typically developing children [13, 14]. Evidence exists that indicates children with disabilities experience higher rates of victimization and negative affect, and lower belongingness at recess [14, 15]. Researchers have also reported evidence that children with developmental disabilities are less physically active at recess than children without disabilities [14, 16, 17]. Children with physical disabilities have been reported to socialize less than peers during free-play and may have difficulty navigating the built environment of the playground, especially children using wheeled mobility [18, 19].

Parents play a crucial role in the lives of their children. For all children, particularly those in traditionally marginalized groups, it is essential for parents to be informed of their child’s educational opportunities [20] as they may need to advocate more for their child’s needs to be met in the school environment. Parents can also serve as an additional informant of children’s experiences at school, which is especially useful when collecting data on children is not feasible such as during a global pandemic. Moreover, the World Health Organization School Policy Framework, which focuses on diet, physical activity, and health, recommends collection of information from and the active involvement of key stakeholders, which includes parents and families [21]. Thus, collecting data on parents’ perceptions of recess all fills a gap in the literature as limited evidence exists on parents’ perceptions of recess in elementary school. The limited data that does exist suggests that parents have a positive view of physical activity during the school day, and that parent views of school-based physical activity are associated with both advocacy for and involvement in school based physical activity opportunities (e.g., recess) [22].

Therefore, the purpose of the current study was to examine parent perceptions of recess, with a specific focus on examining differences by family household income, race and ethnicity, and disability to better understand parents’ views on this critical part of the school day. As it relates to disability, previous research has consistently shown that childhood disability is a stressor for the family unit, has been shown to increase stress and depression in parents, and parental experiences may change as a function of severity [23–25]. As such, rather than examine only whether a child in the home had a disability present, we aggregated the number of disabilities reported across all children in the household as an increasing number of disabilities within the house may differentially affect parent perceptions. Thus, the main research for the current study question was: does disability, family household income, and/or race and ethnicity predict parents’ perceptions of recess? Our hypotheses were that differences in parent perceptions would exist between different groups based on disability status, household income, and the race and ethnicity of the parent.

## Method

All methods and procedures were carried out in accordance with relevant guidelines and regulations governing human subjects’ research.

### Procedures

All study procedures were approved by the Institutional Review Board at the first author’s institution. Data were collected through Prolific [26] (www.prolific.co), an online platform designed for researchers to recruit potential study participants. Previous research has suggested that Prolific produces high quality data that are comparable to, or of a higher quality as compared to other online recruitment methods [27]. Prolific also allows researchers to recruit participants from pre-screened populations. For the current study, we limited the selection of participants to parents. Moreover, in an effort to diversify the sample, participant recruitment was stratified across six household income levels which included annual incomes of <$20,000, $20,000–$44,999, $45,000–$69,999, $70,000–$94,999, $95,000–$119,999, and > $120,000. The survey link was accessible to Prolific user who met these criteria, and each participant received $1.75 USD as remuneration for their participation. Before any data were collected, informed consent was provided by all participants via electronic documentation. Data were collected in May of 2020.

### Participants

Participants in the current study included 473 parents (43% mothers; 33% fathers) from 43 different states within the U.S.A. Reported annual household incomes ranged from <$20,000 (*n* = 36), $20,000–$44,999 (*n* = 72), $45,000–$69,999 (*n* = 96), $70,000–$94,999 (*n* = 99), $95,000–$119,999 (*n* = 74), and > $120,000 (*n* = 96). On average, participants reported having 1.5 children (SD = .77), with an average age of 10 years (SD = 6.1). Forty-two parents reported having a child with a developmental disability, 16 parents reported having a child with a physical disability, and 96 parents reported having a child with social, emotional, or behavioral challenges (e.g., ADHD, depression, anxiety). Finally, 44 parents reported having more than one child with a disability. Table 1 provides an overview of sample demographics.

**Table 1:** Sample descriptive statistics

Descriptive Variable	n (%)
Relation to child	
Mother	218 (45.61)
Father	166 (34.73)
Stepmother	2 (0.42)
Stepfather	2 (0.42)
Grandparent	2 (0.42)
Gender not specified or missing	88 (18.41)
Race/ethnicity	
White	380 (79.66)
African American	24 (5.03)
Asian	34 (7.13)
Hispanic/Latino	28 (5.87)
Hawaiian/Pacific Islander	1 (0.21)
Native American	2 (0.42)
Biracial/multiracial	6 (1.26)
Other	2 (0.42)
Income	
< $20,000	37 (7.76)
$20,000–$44,999	73 (15.30)
$45,000–$69,999	97 (20.34)
$70,000–$94,999	99 (20.75)
$95,000–$119,999	75 (15.72)
>$120,000	96 (20.13)
Disability Status	
Developmental Disability	42 (8.81)
Physical Disability	16 (3.35)
Emotional/Behavioral Disability	96 (20.09)
More than 1 child with disability	44 (9.22)
Child Age	*M* = 10, *SD* = 6.1

### Measures

Demographic data as well as parents’ perceptions of three areas of recess were assessed: belonging and victimization, recess policies, and recess procedures. In completing the full battery, participants were asked to provide information about all children residing in the home. However, in completing the primary measures, participants were instructed to complete these measures considering their youngest elementary school-aged child. All measures are explained in further detail below.

#### Demographics

Demographic information was collected for all participants, including their relation to their children, race and ethnicity, annual household income, and the age and disability status of their children. For child disability status, parents had the option to indicate that the child had more than one disability. All demographic variables were coded as categorical variables and are presented in Table 1.

#### Belonging and victimization

Survey items related to belonging and victimization were based on previous surveys developed and validated by McNamara [15] and colleagues, in which they modified belonging and victimization items from existing scales to be adapted to the recess environment. In the current study, belonging and victimization items were slightly adapted to reflect parents’ perceptions, rather than students (e.g., “I get along well with others during recess” was modified to “my child gets along well with others during recess”). The total measure includes 12 items that represent two sub-scales measuring perceptions of belonging and victimization respectively. All 12 items were measured on a 5-point Likert scale (strongly disagree to strongly agree).

#### Recess policies

Items used to assess parent perceptions of recess policies were adapted from the School Physical Activity Policy Assessment (S-PAPA [28]). This assessment examines policy related to physical activity and recess opportunities at elementary schools and includes three modules: (a) Physical Education; (b) Recess, and (c) Other Before, During, and After School Programs. In the current study, only the Recess module items were used and they were adapted to parent’s report of their beliefs about recess policies. Three sub-scales were utilized in analysis: the importance of recess (e.g., “recess is an important part of the school day”); the unimportance of recess (e.g., “schools should not spend money on recess); and recess withholding (e.g., schools should not be allowed to take away recess for not completing academic work”). All 12 items were measured on a 5-point Likert scale (strongly disagree to strongly agree).

#### Recess procedures

Items used to assess parent perceptions of recess procedures were adapted from the Great Recess Framework – Observational Tool (GRF-OT [29]). The GRF-OT contains 17 items that describe critical aspects of a live recess environment. Previous research has established adequate validity and reliability for this measure. In the current study, 10 items from the GRF-OT were modified to assess two sub-scales: parent reported perceptions of student and staff engagement practices (e.g., “Teachers and staff should encourage a positive culture at recess”) and parent perceptions of the physical environment (e.g., “The recess environment should be free of hazards”). All 10 items (three pertaining to safety and structure, three pertaining to student engagement, four pertaining to teacher engagement) were measured on a 5-point Likert scale (strongly disagree to strongly agree).

### Data analysis

Prior to data analysis, all data were screened for patterns of missingness. Data were also screened for careless responses, with no issues identified. Specifically, data were checked to ensure that all participants responded uniquely to an open-ended question, no participant response time was below two-seconds per item, and no participant response time was below 2 SDs of the mean [30]. To account for missing data present at the item level, models were estimated using Full Information Maximum Likelihood (FIML), based on algorithms implemented in M*plus.* Data were then analyzed using latent variable modeling in M*plus* v8.4 with the weighted least square mean and variance adjusted estimator. As a first step, we used confirmatory factor analysis (CFA) to test the fit of the measurement model of each assessment. Next, we created a household disability index (HDI) to account for how many disabilities each parent reported across all children living in the home. For example, if a participant listed Child 1 with a physical disability and developmental disability, and Child 2 with a physical disability, the corresponding HDI would be a score of “3” for that participant. HDI was then used as a predictor of parent perceptions of recess using a latent variable model framework along with household income and race and ethnicity. Model 1 examined the relationship between HDI, income, race and ethnicity, and parent perceptions of belonging and victimization at recess. Model 2 examined the relationship between HDI, income, race and ethnicity and parent perceptions of importance of recess, un-importance of recess, and recess withholding. Finally, Model 3 examined the relationship between HDI, income, race and ethnicity, and parent perceptions of the physical environment and staff and student engagement at recess.

Decisions about model fit were made using the Chi Square (χ^2^) statistic, the Root Mean Square Error of Approximation (RMSEA), the Comparative Fit Index (CFI), the Tucker Lewis Index (TLI), and the Standard Root Mean Square Residual (SRMR). While the χ^2^ statistic is the most commonly reported measure used in establishing model fit [31], this value is sensitive to sample size, and a non-significant χ^2^ value is often difficult to obtain even when the model is a good fit using other criteria or assessment [32]. As such, it is typical to use model fit indicies that are less dependent on variations in sample size such as the RMSEA, CFI, TLI, and SMRM (Marsh et al., 2004). Cut-off values > .90 for the CFI and TLI have been conisdered indicative of adequte model fit, while values ≥ .95 are preferred for an acceptable modle fit, and cut-off values <.08 have been conisdered indicative of adequte model fit for the SRMR and RMSEA, while values of ≤ .06 for the RMSEA are preferred for an acceptable model fit [32, 33].

## Results

### Victimization and belonging

Results of a CFA revealed that a 2-factor model fit the data in the current study (χ^2^ = 234.52, *p* < .001; CFI = .968; TLI = .959; RMSEA = .098; SRMR = .042). Reliability for each sub-scale was calculated with standardized estimates using McDonald’s [34, 35]. Internal reliability for the belonging sub-scale, as measured by ω was .78. Internal reliability for the victimization sub-scale, as measured by ω was .85. Descriptive statistics for each scale can be found in Table 2. Individual items and factor loadings for the belonging and victimization scale can be found in Table 3. Next, we tested a model that consisted of income, race and HDI and parents’ perception of belonging and victimization, which revealed a fit for the data χ^2^ (70) = 437.63, *p* < .001, RMSEA = .00, CFI = .95, TLI = .94, SRMR = .049. Analyses revealed that parents’ perceptions of their child’s *belonging* at recess were not predicted by income, race, or HDI. Parents’ perception of their child’s *victimization* at recess was not predicted by income or race but was significantly predicted by HDI (*b* = .13, *SE* = .06, *p* = .05). Parents who reported a higher HDI were more likely to indicate that their child experienced victimization at recess. See Table 4 for full results.

**Table 2:** Descriptive statistics for each scale

	N	Median	IQR	Min	Max
Belonging & Victimization					
Belonging	468	20	4	5	25
Victimization	465	11	6	6	30
Recess Policies					
Importance	464	32	6	9	40
Unimportance	478	10	6	0	28
Withholding	466	7	3	2	10
Recess Procedures					
Physical Environment	465	14	3	3	15
Student & Staff Engagement	465	30	5	7	35

Note: *IQR* Interquartile range

**Table 3:** Factor loadings for belonging and victimization scale

Item	Loadings
There are lots of different games my child can play during recess	0.823
My child is threatened at recess by other children	0.873
My child has access to a variety of things to play with at recess	0.811
My child is threatened at recess by adults	0.912
My child has friends they can play with during recess	0.812
My child is not allowed to play with certain groups of children at recess	0.724
My child has been hit, kicked, or scratched at recess	0.715
My child is supported by adults during recess	0.684
My child gets along well with others during recess	0.684
My child has been in a physical fight at recess	0.738
My child is comfortable talking to teachers and staff about problems that happen at recess	0.822
My child has been teased during recess	0.699

**Table 4:** Regression results for belonging and victimization

	Estimate	SE	*P* value
Belonging			
Income	0.031	0.052	0.553
Race	−0.046	0.052	0.381
Household Disability Index	−0.080	0.049	0.102
Victimization			
Income	−0.016	0.048	0.744
Race	0.064	0.048	0.182
Household Disability Index	0.104	0.049	0.032

### Recess policies

Results of a CFA revealed model fit for a 3-factor solution to the data in the current study (χ^2^ = 156.40, *p* < .001; CFI = .986; TLI = .981; RMSEA = .06*7*; SRMR = .031). Internal reliability, as measured by ω, was .89 for recess importance, .84 for recess unimportance, and .74 for recess withholding. Individual items and factor loadings for the parents’ perceptions of recess policies scale can be found in Table 5. The model consisted of income, race, HDI, parent perceptions of recess importance, parent perceptions of recess unimportance, and parent perceptions of recess withholding revealed a fit for the data, χ^2^ (87) = 246.07, *p* < .001, RMSEA = .01, CFI = .98, TLI = .97, SMRM = .037. Regression analyses revealed that income and race were not predictive of parents’ beliefs about the importance of recess, unimportance of recess, or recess withholding policies. Similarly, HDI was not significantly predictive of parents’ perceptions of recess importance or unimportance; it was however significantly predictive of parents’ beliefs about withholding recess. Parents with a higher HDI were significantly more likely to believe that recess should not be withheld from students for behavioral or academic reasons (*b* = .171, *SE* = .07, *p* = .01). See Table 6 for full results.

**Table 5:** Factor loadings for parents’ perceptions of recess policies

Individual Items	Loadings
Recess is an important part of the school day	0.874
Schools should not be allowed to take away recess for behavior problems in the classroom	0.612
Schools should not spend money on recess	0.742
Recess is just as important as any other subject at school	0.799
Children learn important social skills during recess	0.866
Recess is good for children’s health	0.877
Physical activity is not important during the school day	0.787
Schools should be focused on academic achievement, not being physically active	0.801
Play has no place in the school day	0.919
I do not care what happens during recess at my child’s school	0.634
Schools have more important issues to focus on other than recess	0.715
Schools should not be allowed to take away recess for not completing academic work	0.996

**Table 6:** Regression results for recess policies

	Estimate	SE	*P* value
Importance of recess			
Income	0.019	0.055	0.731
Race	−0.001	0.053	0.981
Household Disability Index	0.100	0.057	0.082
Unimportance of recess			
Income	0.036	0.054	0.498
Race	0.004	0.049	0.932
Household Disability Index	−0.082	0.053	0.119
Recess withholding			
Income	−0.078	0.051	0.121
Race	−0.081	0.054	0.134
Household Disability Index	0.129	0.051	0.011

### Recess procedures

Results of a CFA revealed model fit for a 2-factor solution the data in the current study (χ^2^ = 147.89, *p* < .001; CFI = .977; TLI = .969; RMSEA = .085; SRMR = .036). Internal reliability, as measured by ω, was .81 for the physical environment, and .71 for student and staff engagement. Individual items and factor loadings for the parents’ perceptions of recess procedures scale can be found in Table 7. The model that included income, race, HDI, parents’ perceptions of the physical environment, and parent perceptions of student and staff engagement s also demonstrated a model fit, χ^2^ (47) = 103.97, *p* < .001, RMSEA = .043, CFI = .99, TLI = .98, SRMR = .035. Results of the regression analyses revealed that of the demographic variables of focus, only having a higher HDI was predictive of parents’ beliefs about student and staff engagement (*b* = .165, *SE* = .07, *p* = .02). Parents who reported a higher HDI were more likely to agree that recess should be comprised of a variety of activities including physical activity, that teachers should encourage a positive culture at recess while supervising and playing alongside children, and that conflict resolution skills should be taught to children. HDI was not predictive of parents’ beliefs about safety and structure at recess, nor was income or race. See Table 8 for full results.

**Table 7:** Factor loadings for parents’ perceptions of recess procedures

Individual Items	Loadings
Schools should have dedicated outdoor space to recess	0.834
Schools should have grass and natural areas for children to play in during recess	0.889
The recess environment should be free of hazards	0.744
Children should be able to do what they want at recess	
Recess should include a variety of activities for children to play	0.846
Children should be physically active during recess	0.699
Teachers and school staff should encourage a positive culture at recess	0.868
Teachers and school staff should supervise recess	0.752
Teachers and school staff should play alongside children at recess	0.216
Schools should teach children conflict resolution skills during recess	0.546

**Table 8:** Regression results for recess procedures

	Estimate	SE	*P* value
Physical Environment			
Income	−0.033	0.036	0.355
Race	0.047	0.045	0.296
Household Disability Index	0.133	0.079	0.091
Student & Staff Engagement			
Income	0.023	0.035	0.523
Race	0.065	0.041	0.119
Household Disability Index	0.165	0.067	0.015

## Discussion

The purpose of the current study was to gain a better understanding of parents’ views on recess policies and procedures, as well as examine if parents’ perceptions were predicted by three specific factors: HDI, household income, and/or race and ethnicity. Results revealed that parents’ perceptions of recess were predicted by a HDI (household disability index - number of reported disabilities in household, but not income or race and ethnicity. Previous research has documented inequitable access to recess based on race and ethnicity, social-economic status, and disability status [3–6]. However, data in the current study suggests that parental perceptions of recess policies and practices may not differ across household income or race and ethnicity statuses. Yet, for parents of children with disabilities, data in the current study indicate that they perceive greater instances of victimization on the playground, and that they are concerned with reduced access to recess via recess withholding. Limitations to the data in the current study preclude determining if victimization at recess and recess withholding happen more for children with disabilities, and thus are more discussed at home; or if parents are more sensitive to these issues due to exclusion in other parts of the school day [12]. Despite this, these data provide insight to important issues affecting children with disabilities during recess, as well as policy preferences within the schools of parents of marginalized children. The findings also provide insight into parents’ understanding of what happens during recess, which can be an important indicator of recess quality and school adherence to recess policies, which is especially relevant as parents are key stakeholders in the school district [21].

The current findings indicate that parents of children with disabilities report more recess experiences of victimization among their children compared to parents of children without disabilities. This is similar to past research that revealed children with disabilities are at a greater risk of experiencing bullying and victimization during school and at recess [13, 14, 15]. Unsurprisingly, the current findings also indicated that out of all recess procedures, including those regarding physical safety at recess, parents of children with disabilities were most concerned about the social environment. Parents of children with disabilities expressed a greater desire for a recess environment that includes multiple activities, is supervised by adults who also play alongside children, and is a space where conflict resolution occurs. While parents of children with disabilities expressed high agreeability with these recess practices, previous research also supports that these conditions are related to the social and emotional development of children of all abilities [36]. While previous research has focused on the beneficial effect of recess on the physical health of children with disabilities, [16], data in the current study suggest a need to better understand how to support the social and emotional health of children with disabilities during recess and point toward which policies parents find important, but not whether or not these practices are actually happening at recess. Future research should investigate further the degree to which these recess policies are being carried out.

Based on parent perceptions of recess withholding policies in the current study being predicted by parenting a child with a disability, it is possible that recess withholding policies may also be experienced differently for children with a disability. Recess withholding occurs when children are denied access to recess, usually due to academic or behavioral reasons. The current study’s findings that parents’ of children with a disability were more likely to report that recess should not be withheld may indicate that withholding recess is a problem more pertinent to children with disabilities. This would not be surprising, especially considering literature that finds that children with disabilities are more likely to receive exclusionary disciplinary action than children without disabilities [4, 14, 37]. It may also indicate that parents of children with a disability are more aware and sensitive to children being excluded from parts of the school day, especially considering the supporting evidence that children with disabilities have less access to and are more likely to be isolated from these types of social environments [14]. Moreover, the beneficial effects of recess withholding lack evidence and are causing more harm, especially for children with severe behavioral disabilities who would likely benefit from physical activity at recess [4]. Eliminating or reducing recess withholding policies may be beneficial for all children, especially as previous research has revealed strong local district policies on prohibiting recess withholding were associated with schools having increased odds of not withholding recess from students [38].

The current study findings cumulatively highlight the importance of designing and evaluating recess with the needs of children with disabilities in mind. Parents in the present study revealed that this means actions to decrease instances of victimization experienced at recess, ensuring access to recess time, and creating a positive and enjoyable social environment for children with disabilities. It is of utmost importance for school recess to be a safe and stimulating environment for all children, but especially for children with disabilities. Exploration of parents’ perceptions of their children’s recess is an additional avenue to capture children’s experiences at recess that they may not be sharing with teachers or may not be as easily observable on the playground. This is especially relevant since children with disabilities often turn to their parents for support when experiencing bullying [39]. Parents may be important conduits for changing recess policy to better support children with disabilities as they have extensive knowledge in the unique needs of their children and may be situated in a place of leverage to affect necessary change at the school level. Many parents of children with disabilities are already advocating for increased physical activity intervention efforts for their children [16] therefore, gaining a better understanding of their perceptions of how recess can be used as a time for intervention will help researchers create successful evidence-based programs to improve the quality of recess opportunities for all children. Additionally, our results demonstrate that parents of children with disabilities are more likely to support best practices regarding recess compared with parents of children who do not have disabilities. This may be an indication that parents of school children may benefit from parent education on why recess is so critical to kids’ physically, socially, emotionally, and academically, particularly since parents play a powerful role in the policies and practices of districts and schools.

### Limitations & future directions

One limitation of the present study is that children’s disability status was combined into one variable instead of examined separately by specific type of disability, such as physical, intellectual, or emotional/behavioral. Future research should consider examination of differences in parents’ perceptions of recess based on child disability type and not just status. In the present study, only HDI, income, and race and ethnicity as predictors of parents’ perceptions of recess were examined. In the future, researchers should consider investigation of other factors influencing parents’ perceptions of recess such as geographic school location (urban vs. rural [4]). Further, despite race and ethnicity being a variable of interest, the sample lacked adequate representation from many racial and ethnic minority groups, which may have accounted for the null findings reported. We also did not consider other child characteristics such as body size or dual language learning status that have been found to increase chances of experiencing bullying and victimization, future research may benefit from examining such characteristics. Lastly, while data from parents about their children’s experiences at recess provided relevant insights, particularly at a time in which children and teachers were not accessible due to the ongoing COVID-19 pandemic and related school closures, future research that gathers data directly from children, as well as teachers and other school staff who actually engage at recess may provide a more holistic picture of how recess is experienced differently for diverse children.

## Conclusion

Results from this study show that according to their parents, children with disabilities may have a different, and often more negative, experience at recess than children without disabilities. Findings also provide the basis for suggestions that more beneficial recess procedures may be those that teach conflict resolution, provide a variety of engaging activities, and utilize teachers to create a positive environment. School policy makers should begin to look beyond allocation and of recess minutes and add policies that encourage recess quality into legislative reform efforts.

## Data Availability

The dataset used and analyzed during the current study are available from the corresponding author on reasonable request.

## Abbreviations

CFA: Confirmatory Factor Analysis
CFI: Comparative Fit Index
GRF-OT: The Great Recess Framework Observational Tool
HDI: Household Disability Index
RMSEA: Root Mean Square Effort of Approximation
SPAPA: School Physical Activity Policy Assessment
SRMR: Standard Root Mean Square Residual
TLI: Tucker Lewis Index
USA: United States of America
